# Correlative Study of Domain Wall Motion and Pinning at Low Currents in Vortex-like Cylindrical Nanowires

**DOI:** 10.1021/acsnano.6c00987

**Published:** 2026-07-30

**Authors:** Lucía Gómez-Cruz, Núria Bagués, Laura Álvaro-Gómez, Claudia Fernández-González, Ana Arché-Núñez, Joaquín Otón, Marc Raventós-Tato, Lucia Aballe, Eva Pereiro, Aurélien Masseboeuf, Olivier Fruchart, Lucas Pérez, Sandra Ruiz-Gómez

**Affiliations:** † Dpto. Física de Materiales, 16734 Universidad Complutense de Madrid, Plaza de las Ciencias 1, Madrid 28040, Spain; ‡ Alba Synchrotron Light Facility, CELLS, Carrer de la Llum 2-26, Cerdanyola del Vallès, Barcelona 08290, Spain; § Univ. Grenoble Alpes, CNRS, CEA, SPINTEC, Grenoble 38000, France; ∥ 202533 Instituto Madrileño de Estudios Avanzados - IMDEA Nanociencia, C/Faraday 9, Madrid 28049, Spain

**Keywords:** magnetization dynamics, nanowires, correlative imaging, transmission X-ray microscopy, transmission electron microscopy

## Abstract

Cylindrical magnetic nanowires are promising building blocks for next-generation spintronic devices, thanks to their potential for fast and stable domain wall (DW) propagation in three-dimensional architectures. However, energy-efficient propagation remains a major challenge which requires reducing DW drive current and improving control and reproducibility. We demonstrate reproducible Oersted-field-driven DW motion in cylindrical Permalloy (Fe_20_Ni_80_) nanowires with chemical modulations (Fe_70_Ni_30_) at ultralow current density thresholds (*j* = 4.5 × 10^9^ A/m^2^). By tailoring the nanowire geometry to promote azimuthal magnetization, the Oersted field interacts with the magnetic domains, enabling efficient and controlled DW propagation under nanosecond current pulses. Combining transmission X-ray microscopy (TXM) and transmission electron microscopy (TEM), we correlate the magnetic behavior with structural and chemical properties at both designed and unintentional pinning sites. Correlative imaging reveals that unintentional pinning is not associated with compositional changes, but rather with subtle local variations in crystallinity. These results position nanowires in which vortex states can be stabilized as a promising platform for low-power spintronic applications and provide insights into domain wall pinning mechanisms at the nanoscale.

## Graphical abstract

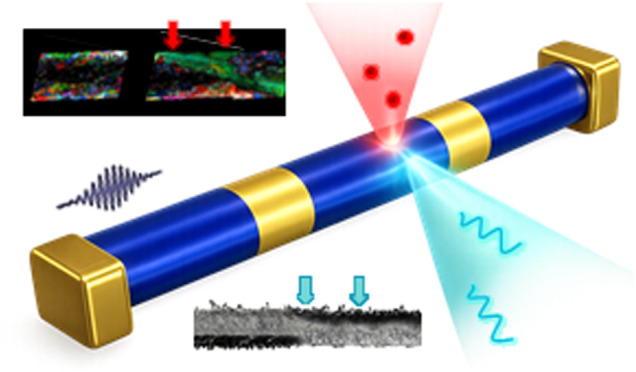

## Introduction

In recent years, the limitations imposed by Moore’s law in electronics, as well as the superparamagnetic effect in conventional magnetic recording, have driven the search for novel device architectures capable of meeting the growing demands for higher data storage densities and processing speeds while simultaneously reducing energy consumption. In this context, innovative nanodevices based on current-controlled domain wall (DW) motion have emerged, offering promising avenues for logic,^1,2^ memory,^3^ and neuromorphic computing^4^ applications. To meet the needs of state-of-the-art information and communication technologies and ensure market penetration of novel devices, it is essential to achieve fast, controllable, reproducible, and energy-efficient DW propagation driven by electric current.

DW-based devices should have reliable control over DW position and their pinning/depinning processes. In general, DW motion occurs when the amplitude of an external magnetic field or electric current exceeds a critical threshold. The energy barrier that a DW must overcome in order to propagate can be deliberately tuned through engineered pinning sites, such as diameter modulations or nanoscale constrictions or protrusions,^5–10^ composition modulations^11–18^ or by controlling the direction of uniaxial crystal anisotropy.^19^ Additional strategies include introducing bends in nanowires with fixed curvature,^20–23^ inducing strain-mediated anisotropy^24^ or applying local ion irradiation.^25,26^ However, DW pinning can also arise unintentionally from factors such as surface roughness,^27–29^ variations in crystalline orientations,^30,31^ local fluctuations in crystallographic anisotropy,^27^ thermal fluctuations^29,32^ or the presence of grain boundaries.^31,33^ Up to now, research on DW pinning has primarily focused on the magnetic response—through the study of depinning fields—or on the global magnetic behavior of the nanosystems, but precise investigations that directly correlate magnetic phenomena with nanoscale structural features are extremely scarce.^34–37^

Among all proposals of magnetic systems, a promising pathway to drastically increase the information density of the devices is the use of three-dimensional and curvilinear magnetic systems.^38–41^ Among these, cylindrical magnetic nanowires have emerged as ideal nanosystems for exploring the potential of 3D nanomagnetism.^42,43^ Cylindrical nanowires provide a unique platform for studying a variety of magnetic textures, including spin textures with azimuthal magnetization components in the domains^17,44,45^ or in the domain walls, like the Bloch point wall (BPW).^46,47^ We have previously shown how pinning sites can be effectively introduced in permalloy nanowires with a strong azimuthal component of magnetization by changing the Fe/Ni ratio in a controlled fashion during electrodeposition.^17,44^

In this work, we explore the critical current density required for DW propagation in this kind of nanowires. Using correlative imaging, combining Transmission X-ray Microscopy (TXM) and Transmission Electron Microscopy (TEM), in the exact same regions of the magnetic nanowires to reveal the causes of unintentional pinning—a key factor, rarely addressed, that limits the minimum current value that can be used for driving DWs through the nanowires. It should be noted that experimental reports on current-induced DW motion in cylindrical nanowires remain scarce, and all of them have been carried out in systems in which magnetization lies parallel to the axis of the nanowires.^48–53^ In contrast, this work is focused on cylindrical nanowires presenting a vortex state with azimuthal magnetization components, in which a prominent role of the current-induced Oe field on the DW dynamics can be anticipated.

## Results and Discussion

Composite images of an electrodeposited permalloy cylindrical nanowire are shown in Figure 1. The Fe_20_Ni_80_ nanowire, of 245 nm diameter and 20 μm length, is deposited and electrically contacted on a Si_3_N_4_ membrane. The NW dimensions are chosen to promote the stabilization of vortex states, in which the vortex core is parallel to the nanowire axis and a strong azimuthal component of magnetization is observed in the magnetic domains. The growth conditions are optimized to obtain a predominantly polycrystalline structure, such that the contribution of magnetocrystalline anisotropy is negligible on average, although local deviations may arise due to variations in grain size and crystallographic orientation, as discussed below. Figure 1a displays high angle (HA) annular dark field (ADF) scanning (S) TEM images (HAADF-STEM) of the full nanowire length, revealing that the diameter is uniform along the entire length, and the surface is smooth, showing no significant surface roughness or microscopic morphological defects such as kinks, constrictions, or discontinuities. This structural uniformity is crucial to reduce the existence of unintentional pinning sites and thus increase the reproducibility of domain wall dynamics.

**Figure 1:**
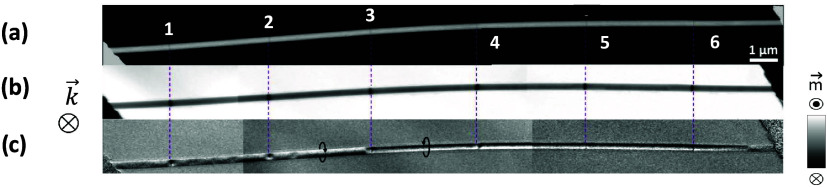
Reconstruction of the Fe_20_Ni_80_ nanowire with chemical modulations (Fe_70_Ni_30_) using stitched (a) HAADF-STEM images, (b) and (c) TXM and XMCD images, respectively. Vertical dashed lines indicate the positions of the chemical modulations. Curved arrows in (c) indicate the azimuthal magnetization directions.

To define specific regions where domain walls can be pinned, a previously reported strategy was implemented involving the incorporation of chemical modulations of different composition (Fe_70_Ni_30_) along the nanowire.^17^ These modulations, each around 60 nm in length, were synthesized by varying the electrodeposition potential during the growth. This technique enables the introduction of sharp compositional changes in selected locations, thereby creating regions with distinct magnetic properties.

In the HAADF-STEM images of Figure 1a—where image contrast is approximately proportional to the square of the atomic number (*Z* ^1.7–2^)—the chemical modulations appear slightly darker than the surrounding Fe_20_Ni_80_ segments due to the relative lower Ni (*Z* = 28) and higher Fe (*Z* = 26) content. These chemically modulated regions, indicated by the purple dashed lines, are even more clearly resolved in the Transmission X-ray Microscopy (TXM) image shown in Figure 1b, acquired at the Fe L_2_ absorption edge. At this energy, the Fe-rich areas absorb strongly and thus appear as darker strips distributed along the nanowire. The modulations are not only well-defined in terms of shape and size but also evenly distributed along the entire nanowire length. This regularity shows the level of precision and reproducibility that can be achieved by means of electrodeposition techniques in the fabrication of nanoscale pinning sites. The direct correlation between HAADF-STEM and TXM allows the unambiguous identification of all modulations, which have each been assigned a unique identifier (number), consistently maintained throughout the manuscript. This approach enables tracking individual pinning sites and identifying their role in domain wall dynamics.

In addition to transmission images, synchrotron-based TXM allows the acquisition of dichroic images by measuring the pixel-by-pixel difference between images taken with opposite X-ray helicities. The contrast of such X-ray magnetic circular dichroism (XMCD) images is indicative of the magnetization component along the X-ray propagation direction, providing thus access to the magnetic configuration of the nanostructure. A schematics of the geometry of the TXM measurements can be found in the Supporting Information (Figure S1). Due to vortex state of the nanowires, the magnetic contrast varies from bright (dark) at the top to dark (bright) at the bottom, depending on the circulation direction—positive or negative—of the magnetic domains. Figure 1c shows the XMCD image of the same nanowire measured at θ = 0° (i.e., X-ray incidence perpendicular to the wire axis). The segments of the nanowire between the left contact and modulation 3 exhibit all similar magnetic contrast: the top parts appear bright, indicating a magnetization component antiparallel to the photon wavevector *k⃗*, i.e., pointing outward from the image. The bottom parts of these segments show a dark contrast, which indicates magnetization parallel to *k⃗*, i.e., pointing inward. The magnetization in this region is thus azimuthal, with curling sense indicated by the black curved arrow. In contrast, the segments from modulation 3 toward the right contact display the opposite configuration, indicating the presence of a domain wall at modulation 3, separating two magnetic domains with opposite azimuthal magnetization. Domains characterized by a dark top and bright bottom contrast are referred to as having negative circulation, while those with bright top and dark bottom are labeled as having positive circulation. It is important to note that the chemical modulations also exhibit azimuthal states with a defined sense of circulation. This configuration reduces the demagnetizing field arising from the magnetization mismatch at the modulation interfaces.^18^ In Figure 1c, all chemical modulations except modulation 3 show negative circulation (dark top and bright bottom), which matches the long segment circulation on the right-hand side of the nanowires and is opposite to that of the left-hand side domain.

After confirming that the nanowires exhibit a vortex magnetic state and that domain walls can be nucleated and pinned on chemical modulations, we systematically studied the current-induced domain wall motion. Starting from a random initial magnetic state, current pulses of fixed duration (2 ns) and varying amplitude were applied between every two XMCD-TXM images. When the current flows toward the left (right) contact, we refer to it as left (right) polarity. The resulting Oersted field favors domains with positive (negative) circulation when current pulses of right (left) polarity are applied, as illustrated in Figure S1 (see Supporting Information).

We first investigated the possibility to obtain a reproducible DW motion between chemical modulations. The initial magnetic state shown in Figure 2b­(i) displays a domain wall located at modulation 5. After a single current pulse with amplitude *j* = 6.5 × 10^10^ A/m^2^ and left polarity (i.e., with an associated Oe field parallel to the azimuthal domains with negative circulation) the NW magnetic configuration changes to the state shown in Figure 2b­(ii). The domain wall has shifted two modulations to the left, reaching modulation 3. This motion expands the domain with negative circulation, in alignment with the Oe field direction.

**Figure 2:**
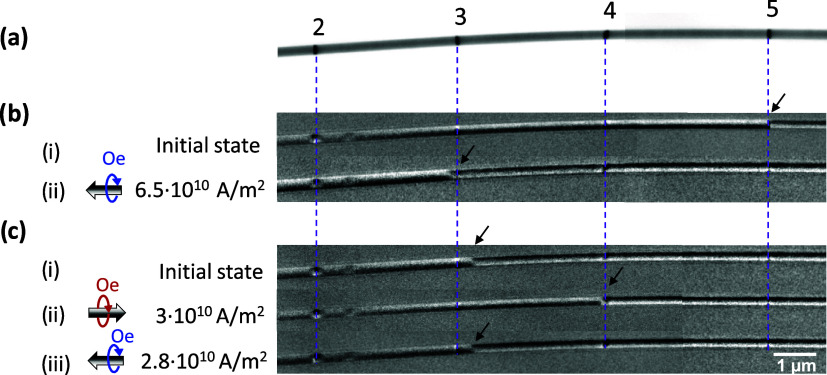
Transmission X-ray imaging of domain wall motion between modulations under the application of current pulses. (a) Transmission image at the Fe L_2_ edge, with the purple dashed lines indicating the positions of the chemical modulations. (b) (i and ii) and­( c) (i–iii) Sequences of XMCD-TXM images, taken before and after applying 2 ns current pulses with different polarities and current densities (indicated by the left sketches). The DW positions in the images are marked with black arrows.

Given that the domain wall overcomes two consecutive chemical modulations at this current density, we reduced the current further and reversed the pulse polarity to test whether domain wall motion between adjacent modulations could be achieved at even lower amplitudes. We started from an initial state shown in Figure 2c­(i), with a domain wall pinned near modulation 3. It should be noted that, in this case, the domain wall is not pinned exactly at the center of the modulation but slightly to the right. After applying a current pulse with an amplitude of *j* = 3 × 10^10^ A/m^2^ and right polarity, the domain wall moved one modulation to the right, reaching modulation 4 (Figure 2c­(ii)). Notably, this current density is less than half of the value used in panel (b). Applying a similar pulse, *j* = 2.8 × 10^10^ A/m^2^, but with the opposite polarity, we moved the domain wall back to its original position (see Figure 2c­(iii)), confirming the reversibility of the motion between chemical modulations. This reproducible motion was checked for a total of 10 movements spanning over different modulations along the nanowire, with similar current densities in all of them.

This repeatable and directional movement demonstrates controlled Oe-field-driven domain wall motion under nanosecond current pulses, with the chemical modulations acting as effective pinning sites. In addition, the minimum current density to induce controllable motion between two adjacent modulations, *j*_thr,1_ = 2.8 × 10^10^ A/m^2^, is more than 5 times lower than previously reported in the literature.^54^ The threshold current densities reported in the literature to drive domain wall motion in cylindrical nanowires made of different ferromagnetic alloys, as well as in planar permalloy nanostripes are summarized in Table 1. It should also be noted that we use 4 times shorter pulses than Bran et al.,^54^ where domain walls were propagated in modulated Ni cylindrical nanowires. Two important remarks can be extracted from the comparison of the experiments reported in this work and the ones in the cited literature. On the one hand, there is a clear reduction of the threshold current density when moving from planar (2D) to cylindrical structures, highlighting the importance of three-dimensional magnetization configurations, where the magnetization is no longer confined to a two-dimensional plane. On the other hand, similarly to what happens in planar systems, permalloy appears to be the best option also in cylindrical systems for energy-efficient domain wall motion. At the employed current densities considerable impacts of spin-transfer torque or Joule heating on magnetization dynamics can be excluded. In fact, we have studied more than 200 events of DW motion under current pulses and, in all cases, the movement was in the direction of the Oe field, irrespective of the direction of the spin-transfer torque (see figure S3). Therefore, we can conclude that, in these nanowires, the Oe field is the dominating driving force.^55^

**Table 1:** Experimental Observation of Current-Driven Domain Wall Motion in Different Cylindrical Nanowires as well as in Permalloy Planar Nanosystems

**material**	**diameter (nm)**	**current density (A/m^2^)**	**refs**
FeNi (present work)	245	4.5 × 10^9^	
CoNi (multisegmented) (^a^)	80	6 × 10^10^	^56^
Co_30_Ni_70_	90	1.2 × 10^12^	^48^
Ni	100	1.5 × 10^11^	^54^
Py (nanostrip)	300 nm width	1.5 × 10^12^	^57^
Py (curved nanostrip)	960 nm width	1 × 10^12^	^58^

a In this system, domain wall motion is assisted with a magnetic field of 31.5 mT.

For a uniform current density, the magnitude of the Oe field inside a cylindrical NW, at a radial distance *r* from the center, can be directly derived from the Ampere’s law, and it is given by *H* = *jr*/2. For our nanowire with a radius of approximately 120 nm, the current density j_*thr*,1_ corresponds to approximately *H*_thr,1_ = 1.7 × 10^3^ A/m (2.1 mT) at the surface.

Even though the NWs appear highly homogeneous in terms of diameter, roughness, and composition on the scale of tens of nanometers, a closer examination is necessary to fully understand the origin of domain wall pinning. Therefore, the quality of the chemical modulations was analyzed using energy-dispersive X-ray spectroscopy (EDS) mapping combined with ADF-STEM to visualize chemical and structural variations within and around the modulations. Although the growth conditions are nominally the same for all modulations, we have found two different types in the nanowires. The first and most prevalent type encompasses modulations 1, 2, 3, and 6; here, modulation 3 (shown in Figure 3a–c) serves as the representative example. The second, less frequent structural variant includes modulations 4 and 5, with modulation 4 (in Figure 3d–f) acting as the representative case.

**Figure 3:**
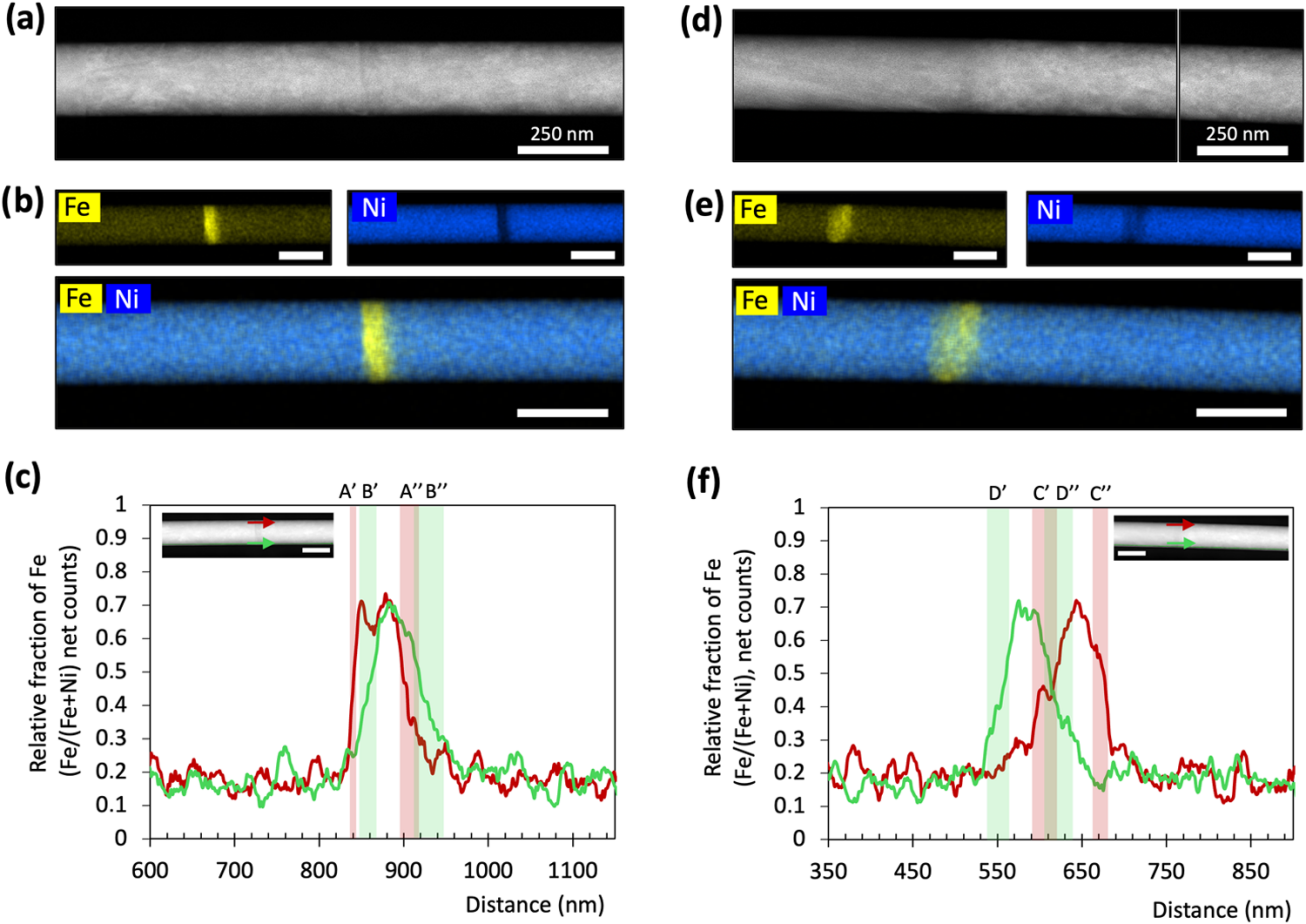
ADF-STEM images and EDS maps of two types of modulations represented by modulation 3 (a–c) and modulation 4 (d–f). Panels (a and d) are the ADF-STEM images, panels (b and e) display the Fe and Ni elemental maps along with a composite Ni + Fe intensity map, and panels (c and f) present relative elemental profiles of Fe across the top (red) and bottom (green) edges of the nanowire, focused on the modulation regions to show the interface widths, which are highlighted with semitransparent boxes. Boxes A and C (red) correspond to the top edge profiles, whereas B and D correspond to the bottom edge profiles. The single (′) and double (″) primes denote the left and right interfaces of each modulation, respectively. The line profiles were obtained from the Fe intensity (counts) of the EDS maps and normalized using the ratio Fe/(Fe + Ni).

For the case of modulations similar to modulation 3, they present a width of around 60 nm, with interfaces almost perpendicular to the edge of the nanowire—tilted by about 4°. In the ADF-STEM image (Figure 3a), the contrast appears similar on both sides of the modulation. Figure 3b shows the Fe and Ni net intensity maps across the modulation region, clearly indicating the position of the modulation. The relative elemental profile of Fe across the top and bottom edges of the nanowire (Figure 3c) reveals that the Fe fraction is approximately 0.18 ± 0.02 before (left) and 0.17 ± 0.02 after (right) the modulation, while it increases to 0.67 ± 0.02 at the modulation point. The widths of the modulation interfaces were measured from the line profiles shown in Figure 3c, using the 25–75% composition drop criterion. These measurements reveal a chemical asymmetry between the two sides or the modulation: the left interface is sharper, whereas the right interface is significantly more diffuse, extending several nanometers into the adjacent region. As summarized in Table 2, and show in Figure 3, the left interface is sharper with widths of 8 nm (top, A′) and 22 nm (bottom, B′), while on the right the interface widths are 24 nm (top, A″) and 39 nm (bottom, B″). However, these values should be considered as upper estimates of the actual interface widths, as they may be influenced by local fluctuations in Fe and Ni concentrations through the nanowire thickness, as well as by potential tilting of the interfaces relative to the electron beam direction.

**Table 2:** Interface Width Values for Modulation 3 and Modulation 4^a^

		**Interface width**			
		**left**		**right**	
**modulation 3**	top edge	A′	8 nm	A″	24 nm
	bottom edge	B′	22 nm	B″	39 nm
**modulation 4**	top edge	C′	32 nm	C″	23 nm
	bottom edge	D′	27 nm	D″	37 nm

a The letters correspond to the boxed areas indicated in Figure 3.

The ADF-STEM image of modulation 4 (Figure 3d) shows a contrast change between the two sides of the modulation, suggesting possible structural and/or chemical changes. The Fe and Ni net intensity maps for modulation 4 (Figure 3e) indicate a modulation width between 100 and 120 nm, with interfaces tilted by approximately 12° relative to the nanowire axis. In this case, there are also visible variations in the Fe and Ni net intensities within the modulation region itself, suggesting local fluctuations in the relative Fe and Ni concentrations or a possible inclination of the interfaces along the viewing (projection) direction. The relative elemental profiles extracted from the top and bottom edges of the nanowire (Figure 3f) reveal similar Fe concentrations as observed in Figure 3c. Modulation 4 exhibits more diffuse interfaces than modulation 3. The measured interface widths are shown in Table 2 and in Figure 3f, and in all cases are equal to or exceed 23 nm (C′, C″, D′, D″).

We have seen in Figure 2c, as well as in different experiments described throughout the manuscript and in the Supporting Information that, in several cases, domain walls are pinned outside the chemical modulations. However, there is no evidence of strong inhomogeneities in either the structure or chemical composition that could justify this effect. Although small differences are observed between the two interfaces of the chemical defect—with one interface being better defined and more perpendicular to the NW axis than the other, as discussed above—the resulting variations in local magnetocrystalline anisotropy, magnetoelastic anisotropy, and saturation magnetization are negligible compared to the magnetostatic interactions between the magnetic charges associated with a domain wall^59^ and those of the chemical modulation. Furthermore, the structural asymmetries resolved near these chemical barriers are smaller than the expected domain wall width in permalloy and therefore should not lead to asymmetric pinning. Therefore, the pinning of domain walls close to, but not exactly at, the modulation is most likely governed by these long-range magnetostatic interactions, rather than by local structural or compositional variations. Similar interactions have recently been observed in thinner permalloy nanowires with chemical modulations and longitudinal magnetization.^60^ Furthermore, the differing magnetization distributions in this scenario (longitudinal vs azimuthal) necessitate a more thorough investigation.

Once identified the minimum current required for reproducibly propagating domain walls between two chemical modulations, we further reduce the current in order to investigate the absolute threshold for domain wall motion. We have explored current densities below *j*_thr,1_ = 2.8 × 10^10^ A/m^2^, starting from initial states in which a domain wall is pinned at a chemical modulation. In Figure 4, we present some representative results. Additional data can be found in the Supporting Information (Figure S2).

**Figure 4:**
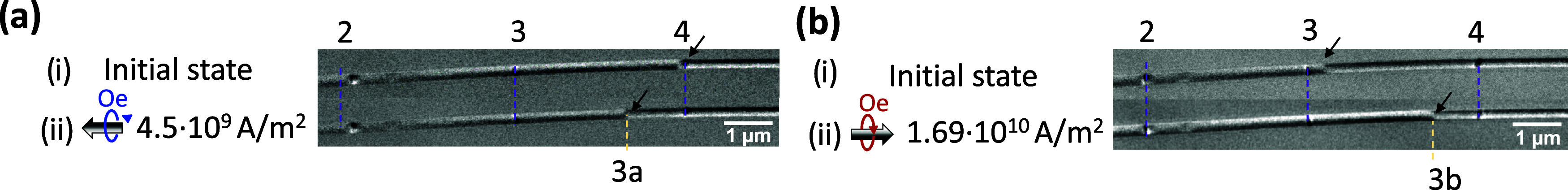
XMCD-TXM images of domain wall motion in between the modulations. Panels (a, b) (i) show the initial magnetic state, and (a, b) (ii) the resulting state after applying the current pulse with density and polarity indicated in the image.

Starting with a domain wall pinned at modulation 4 (Figure 4a­(i)), a current pulse with *j* = 4.5 × 10^9^ A/m^2^ and left polarity was applied, which favors the domain with negative circulation. As shown in Figure 4a­(ii), the domain wall moves toward the left, but does not reach the adjacent modulation 3, stopping at position 3a. In Figure 4b we illustrate a similar behavior; the domain wall moves to the right applying a current pulse with right polarity of *j* = 1.69 × 10^10^ A/m^2^, but stops before reaching the next modulation 4, stopping at position 3b. We have found a minimum current density capable of inducing domain wall motion of *j*_thr,2_ = 4.5 × 10^9^ A/m^2^. This amplitude is, to the best of our knowledge, about 30 times lower than the lowest current densities reported so far.

This observed threshold current density (*j*_thr,2_ = 4.5 × 10^9^ A/m^2^) corresponds to an Oe field of 270 A/m (0.34 mT) at the nanowire surface. Although low, this value is larger than the one expected for field-driven domain wall motion, pointing to the existence of unintentional pinning sites in the nanowire, i.e., defects other than the chemical modulations intentionally introduced. In fact, several intermediate positions between the modulations were identified as recurring pinning points in experiments with ultralow current. Since no microscopic inhomogeneities were observed in the nanowire shape or composition, which could be responsible for the DW pinning, we performed a detailed correlative characterization of the NW composition and crystalline structure in the vicinity of the unintentional pinning sites, using the chemical modulations as reference points for colocalization. Figure 5 presents a structural and chemical analysis of the two pinning locations shown in Figure 4, labeled 3a (Figure 4a­(ii)) and 3b (Figure 4b­(ii)). According to TXM data, these pinning sites are located between modulations 3 and 4, at around 1.23 and 0.92 μm from the left side of modulation 4, respectively. Figure 5a,b shows the location of each pinning site 3a and 3b on the ADF-STEM images thanks to the measured TXM distances.

**Figure 5:**
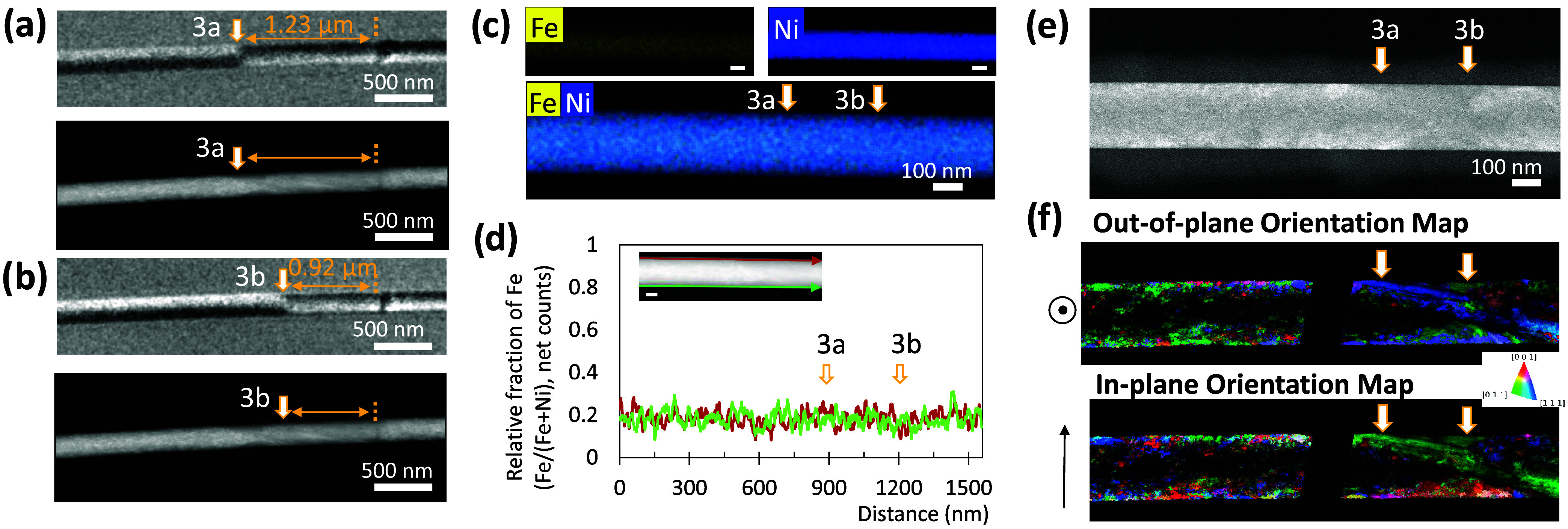
XMCD-TXM (top) and the ADF-STEM (bottom) images of regions (a) 3a and (b) 3b. (c) Fe and Ni EDS elemental maps along with a composite Ni + Fe intensity map. (d) Ni/Fe line profile along the nanowire. (e) Lower angle ADF-STEM image containing both pinning sites. (f) Presents crystal orientation maps for regions 3a and 3b, showing both in-plane (bottom) and out-of-plane (top) orientations. The in-plane orientation is defined relative to the image’s vertical axis, which is perpendicular to the nanowire.

The EDS map (Figure 5c,d) does not indicate any significant chemical variation. Line profiles along the length of the wire show a uniform relative fraction of Fe and Ni, allowing us to conclude that the unintentional DW pinning is unlikely to be associated with substantial compositional changes along the nanowire. ADF-STEM images encompassing both pinning sites were acquired at lower collection angles to enhance crystallographic contrast and reveal structural details. In the ADF-STEM image, Figure 5e, both pinning regions appear predominantly polycrystalline. However, subtle contrast variations in the vicinity of the pinning sites may indicate the presence of larger grains. Therefore, a more detailed crystallographic analysis is needed to establish whether the observed pinning has structural origin.

We performed nanobeam electron diffraction (NBED) in the 4D-STEM mode in these regions. The diffraction data sets were used to obtain crystal orientation maps using automatic crystalline orientation mapping (ACOM)^61^ and procedures described in the experimental section. From these data sets, we generated out-of-plane (top) and in-plane (bottom) crystal orientation maps for each pinning region, as presented in Figure 5f. The center of the nanowire appears dark due to the limited diffraction signal caused by the increased sample thickness in the middle of the wire, but structural details remain visible at the edges. A large grain (green in the in-plane crystallinity map), corresponding to a grain oriented along the [110] direction, is observed. A preferred texture in this direction has been previously observed in permalloy nanowires fabricated under similar conditions in membranes with smaller pore diameters. This grain spans region 3b and may act as a potential source of domain wall pinning. Pinning site 3a is located at the boundary between this large grain and a region with a powdery structure composed of randomly oriented crystals. This local variation in crystallinity within the nanowire can act as a pinning site for domain walls, increasing the current density required to drive domain wall motion and thereby affecting the reproducibility of the motion. Although the pore diameter of the membranes and the growth conditions were selected to promote the formation of polycrystalline nanowires, local variations in the crystallinity can still arise. Improving the growth process to suppress such local crystallographic variations could potentially reduce the current required for domain wall motion, paving the way for devices with lower energy consumption.

## Conclusions

In conclusion, we demonstrate reproducible domain wall motion in cylindrical Fe_20_Ni_80_ nanowires with engineered Fe_70_Ni_30_ chemical modulations, driven by nanosecond current pulses and mediated by the Oe field. Domain wall propagation occurs with high reproducibility between modulations, exhibiting a minimum threshold current density of *j* = 2.8 × 10^10^ A/m^2^, significantly lower than previously reported values in similar nanostructures. The chemical modulations - controlled compositional changes introduced during electrodeposition - serve as reliable and reproducible pinning sites. Furthermore, domain walls can also become pinned at intermediate positions in the absence of chemical modulations at lower current densities, specifically at *j* = 4.5 × 10^9^ A/m^2^. Correlative imaging by transmission X-ray microscopy and transmission electron microscopy reveal that this unintentional pinning sites are not related to compositional inhomogeneities but are instead associated with local changes in the crystalline structure. Our results highlight the potential of Oersted-field-driven dynamics in nanowires with vortex magnetic states and underscore the importance of understanding the origin of pinning to further reduce the DW motion current threshold.

## Methods

### Sample Fabrication and Preparation

Permalloy (Fe_20_Ni_80_) cylindrical nanowires with a diameter of 245 nm and composition modulations (Fe_70_Ni_30_) along their length were synthesized via template-assisted electrochemical deposition. Nanoporous anodic aluminum oxide (AAO) templates were prepared by one-step hard anodization of Al disks (Goodfellow, 99.999% purity), which were previously electropolished in a perchloric acid and ethanol solution (1:3) at 20 V for 5 min at 3 °C. Anodization was carried out in an aqueous solution of oxalic acid (0.3 M) and ethanol (0.9 M), applying 140 V at 0–1 °C for 2.5 h. The remaining Al was etched using an aqueous solution of CuCl_2_ (0.74 M) and HCl (3.25 M), and the pores were widened to a final diameter of 245 nm using 5 vol% H_3_PO_4_.

Before electrodeposition, a thin Au film was thermally evaporated on one side of the membrane to serve as a working electrode. The electrolyte consisted of NiSO_4_ (0.47 M) and NiCl_2_ (0.01 M) as Ni^2+^ sources, FeSO_4_ (0.09 M) as the Fe^2+^ source, and H_3_BO_3_ (0.4 M). All chemicals were dissolved in deionized water, and the pH was adjusted to 2.3 using 10 vol % H_2_SO_4_. The nanowires were grown at room temperature (21–25 °C) by alternating the applied voltage between *V*_1_ = −1.35 V and *V*_2_ = −1.0 V (vs Ag/AgCl) in a three-electrode electrochemical cell using a PGSTAT potentiostat (Metrohm-Autolab), with a Pt mesh as the counter electrode and an Ag/AgCl (3 M NaCl) reference electrode. Long and short pulses were alterned to grow 3.6 μm Fe_20_Ni_80_ segments and 60 nm Fe_70_Ni_30_ modulations, respectively.

Due to the anomalous codeposition effect shown by Fe and Ni, the composition of electrodeposited FeNi alloys strongly depends on growth potential as well as on the geometry of the working electrode,^62^ whereas the crystalline structure depends on the growth rate. The electrochemical system was calibrated to obtain the desired composition (Fe_20_Ni_80_) for large overpotentials (*V*_1_ = – 1.35 V), in the region in which the electrodeposition is controlled by mass-transport. Under these conditions, a large growth rate of 17 nm/s is reached and a polycrystalline structure is expected. For the growth of the chemical modulations, the potential is reduced to *V*_2_ = −1.0 V, reducing the growth rate to 3 nm/s. It is important to note that the electric circuit is physically opened between each deposition step, ensuring that no current flows through the sample and providing sufficient time for the electrochemical system to recover equilibrium conditions. Using this method, structurally defect-free nanowires were successfully obtained. In addition, the reduced growth rate provides better control over the length of the chemical modulations. This nanowire design results in segments that enable fast DW propagation, separated by chemical modulations at controlled distances where domain wall pinning is expected to occur.

Following growth, the Au layer was removed with a 0.1 M I_2_/0.6 M KI solution, and the alumina template was dissolved in a solution of 0.4 M H_3_PO_4_ and 0.2 M H_2_CrO_4_. The nanowires were then dispersed onto Si substrates with 20 nm thick Si_3_N_4_ windows to enable X-ray and electron transmission microscopy experiments. For electrical excitation using nanosecond current pulses, the nanowires were contacted via laser lithography at the Centre of Micro and Nanofabrication of IMDEA Nanoscience. The measured resistivity was approximately 14.8 μΩ·cm.

### Transmission X-ray Microscopy

XMCD-TXM measurements were performed at the MISTRAL beamline of the ALBA Synchrotron Light Facility in order to simultaneously access the chemical composition and three-dimensional magnetization texture of the samples. We measured at the Fe L_2_ absorption edge energy (721.7 eV) using both right- (C+) and left-handed (C−) circular polarizations. XMCD images were obtained by the pixel-wise subtraction of images obtained with opposite polarizations. Nanosecond current pulses were injected through the sample using a specially designed sample holder equipped with a printed circuit board (PCB) to contact the sample. The pulse generator utilized was an AVTECH AVR-E Series.

### Transmission Electron Microscopy

Structural and chemical analysis of the nanowires were performed using a double-aberration-corrected Thermo Fisher Scientific Spectra 300 (S)­TEM operated at 300 kV, and equipped with a Super-X EDS detector. The convergence angle for STEM imaging and EDS mapping was set to 19.5 mrad. To improve the signal-to-noise ratio (S/N) of the EDS-STEM data, the spectrum imaging maps were prefiltered (9 px average) before extracting Ni and Fe net intensity maps. The interface width was determined using the 25–75% composition drop criterion, which is defined as the distance between the points where the composition decreased from 75 to 25% of the total change across the interface. STEM images were acquired in HAADF, medium-, and low-angle ADF modes. The inner and outer collection angles were 63 and 200 mrad for HAADF, and 31 and 188 mrad for medium-angle ADF, in both cases using the HAADF detector. Low-angle ADF images were acquired using a DF-S Panther STEM detector with a full angular range of 15–56 mrad; only the outer ring of this detector was used for image acquisition to reduce direct beam contribution. These imaging conditions were selected to enhance different types of contrast: HAADF primarily for chemical (*Z*-contrast), medium-angle ADF (referred to as ADF in the main manuscript) for a mix of chemical and structural (diffraction) contrast, and low-angle ADF mainly for structural contrast. For visualization purposes, contrast adjustments were applied to the medium-angle ADF-STEM images. Nanobeam electron diffraction (NBED) mapping in 4D-STEM mode was collected from targeted regions using a convergence angle of 1 mrad and a pixelated EMPAD detector.

To perform Automatic Crystalline Orientation Mapping (ACOM),^61^ the microscope was calibrated using a NBED data set from a standard AuPd calibrant (50% Au–50% Pd, FCC, *a* = 0.398 nm). An excitation error tolerance of 0.03 Å^–1^ (standard deviation) was applied during template matching. Calibration was performed with the py4DSTEM software package,^63^ by identifying diffraction peaks through cross-correlation with simulated templates. The same Bragg peak detection procedure was then applied to the NBED data set from the nanowire sample to extract experimental peak positions. Given that the crystal parameters of the material are well established (75% Ni–25% Fe, FCC, *a* = 0.352 nm), we generated a library of simulated orientations and diffraction patterns. The orientation space was sampled with steps of 2° for out-of-plane rotations and 5° for in-plane rotations. This sampling yielded a total of 406 unique orientations, reflecting the high symmetry of the cubic system. In such systems, the fundamental orientation space is restricted to a 90 × 45° sector bounded by the [001], [011], and [111] directions.

## Supplementary Material



## Notes

Schematics of the XMCD-TXM setup, additional experiments on domain wall motion, a TEM study of an additional region (close to modulation 6) (PDF)

## ⊥

Synchrotron SOLEIL, L’Orme des Merisiers, Saint-Aubin 91190, France
