# Supplementary material for: Correlative Study of Domain Wall Motion and Pinning at Low Currents in Vortex-like Cylindrical Nanowires
Source: ACS Nano. 2026 Jul 30;20(31):21615–24. doi: 10.1021/acsnano.6c00987 (PMC13472331; doi:10.1021/acsnano.6c00987)
Supplement: Supplementary file 1 [file nn6c00987_si_001.pdf]

Supplementary Information
Correlative Study of Domain-Wall Motion and Pinning at Low Currents in Vortex-Like Cylindrical Nanowires

Lucía Gómez-Cruz,[†,§] Núria Bagués,[‡] Laura Álvaro-Gómez,[†] Claudia Fernández-González,[‡] Ana Arché-Núñez,[‡] Joaquín Otón,[‡] Marc Raventós-Tato,[‡] Lucia Aballe,[‡] Eva Pereiro,[‡,‖] Aurélien Masseboeuf,[¶] Olivier Fruchart,[¶] Lucas Pérez,[*,†,⊥] and Sandra Ruiz-Gómez[*,‡]

†*Dpto. Física de Materiales, Universidad Complutense de Madrid, Plaza de las Ciencias 1, Madrid, 28040, Spain*

‡*Alba Synchrotron Light Facility, CELLS, Carrer de la Llum 2-26, Cerdanyola del Vallès, 08290, Barcelona, Spain*

¶*Univ. Grenoble Alpes, CNRS, CEA, SPINTEC, 38000 Grenoble, France*

§*Univ. Grenoble Alpes, CNRS, CEA, SPINTEC, 38000 Grenoble, France*

‖*Current address: Synchrotron SOLEIL, L'Orme des Merisiers, 91190 Saint-Aubin, France*

⊥*Instituto Madrileño de Estudios Avanzados - IMDEA Nanociencia, C/ Faraday 9, Madrid, 28049, Spain*

E-mail: lucas.perez@ucm.es; sruiz@cells.es

Transmission X-Ray Microscopy

Figure S1 shows the schematic of the setup used for imaging magnetism via transmission X-ray microscopy (TXM). Transmitted images were acquired at the Fe L_2 absorption edge (721.7 eV) to enhance magnetic contrast both at the modulations (Fe-rich) and in the rest of the nanowire (Ni-rich), using right-handed (C^+) and left-handed (C^-) circularly polarized light.

Due to the azimuthal magnetization of the nanowires, the magnetic contrast varies from white (black) at the top to black (white) at the bottom, depending on the circulation direction—positive or negative—of the magnetic domains.

The spatial resolution is approximately 10 nm/pixel. Nanosecond current pulses were applied to the sample between each set of images to promote domain wall motion. The resulting Oersted field favors domains with positive (negative) circulation when current pulses of right (left) polarity are applied, as illustrated in the figure.

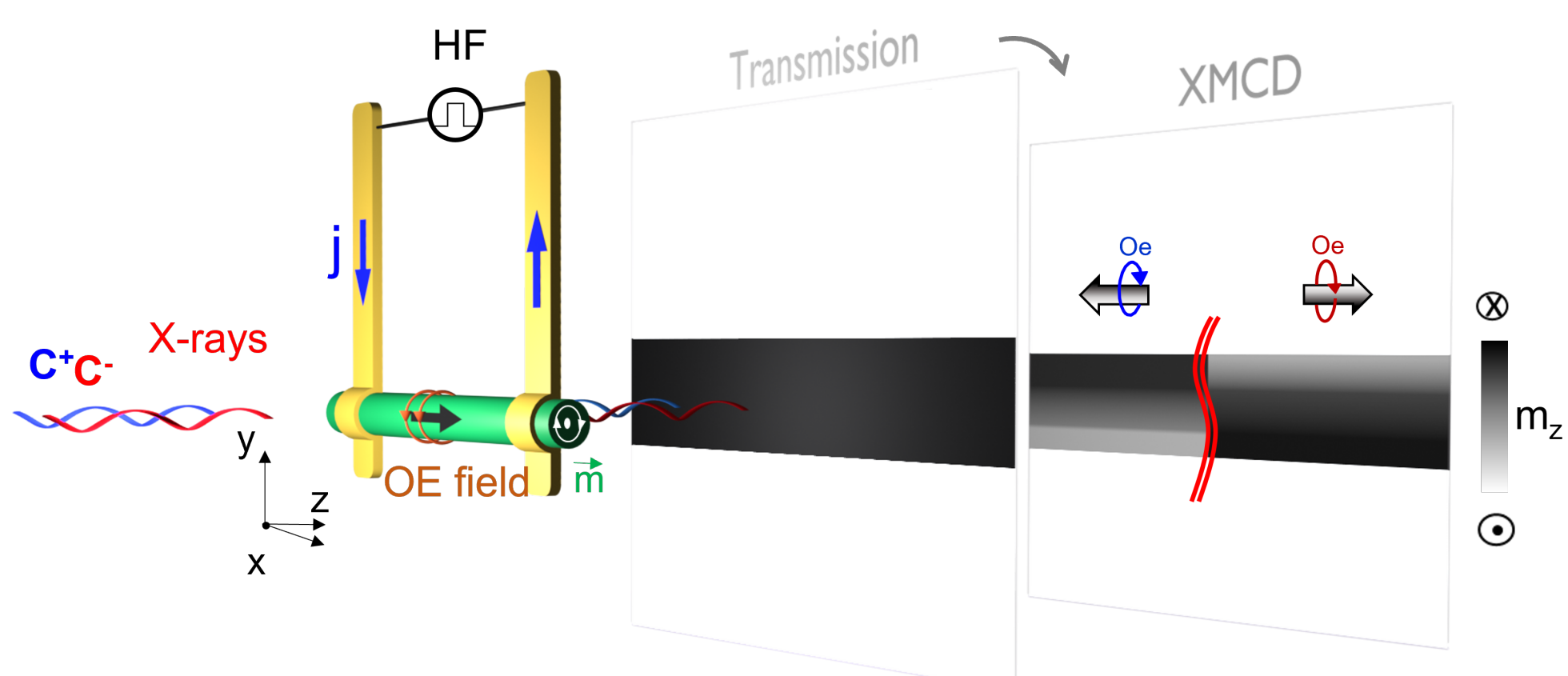

Figure S1: Schematic representation of the set up for obtaining XMCD images via Transmission X-Ray Microscopy.

Single Domain wall motion

Figure S2 shows additional domain wall motion events under current pulses. In all cases, the motion favors domains with the same circulation as the Oersted field generated by the current. The applied current density remains below the threshold required to drive domain wall motion between the modulations ($j_{thr,1} = 2.8 \times 10^{10}$ A/m^2), resulting in intermediate domain wall pinning positions.

Several events are presented, showing different initial and final magnetic states, with variations in both current density and polarity. In Figures S2(a–c), the observed pinning positions correspond to those discussed in the main manuscript—namely, pinning sites 3a, 3b, or their immediate vicinity. Figures S2(d–e) show similar events occurring in other regions of the nanowire.

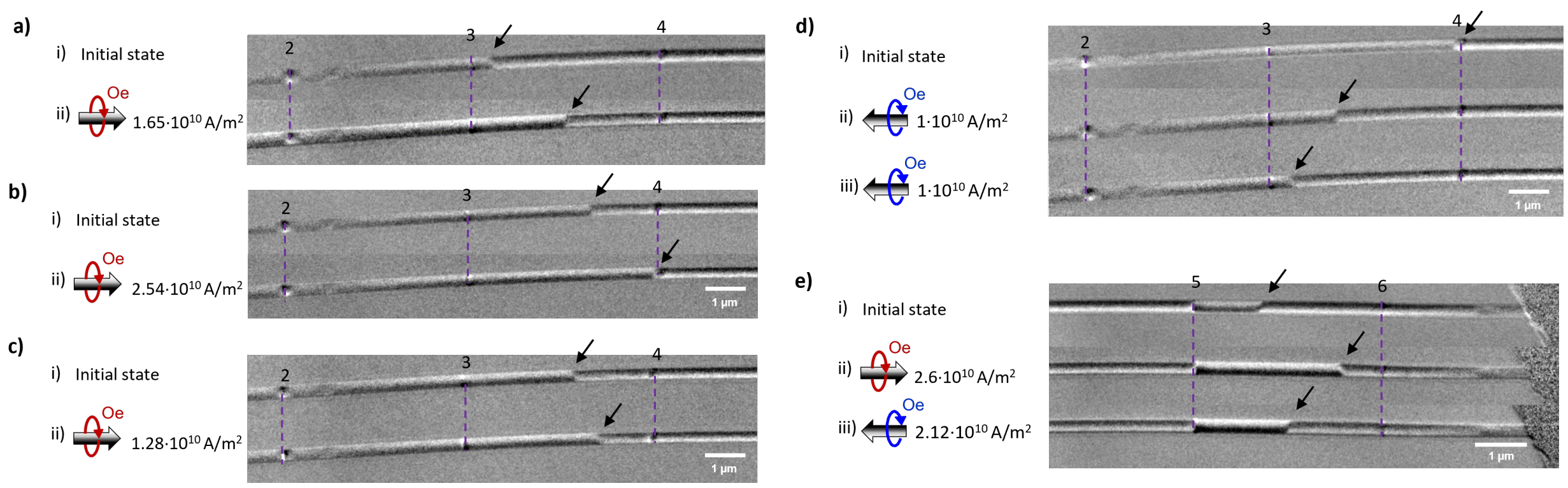

Figure S2: XMCD-TXM images of domain wall motion under 2ns current pulses between modulations 3 and 4 (a-d) and modulations 5 and 6 (e). The polarity and density of the current pulses are indicated in the figure.

Multiple Domain walls motion

Figure S3 shows additional domain wall motion events in a nanowire where multiple domain walls have been initialized. In all cases, the motion favors domains with the same circulation as the Oersted field generated by the current, with the domain walls marked by a yellow asterisk moving in the direction opposite to that expected from STT. Overall, the domain

walls follow the direction set by the Oe field, leading to an increase in the size of domains whose circulation matches that of the Oe field. This results in the expansion of white-top/black-bottom domains in panels (a) and (b), and black-top domains in panel (c).

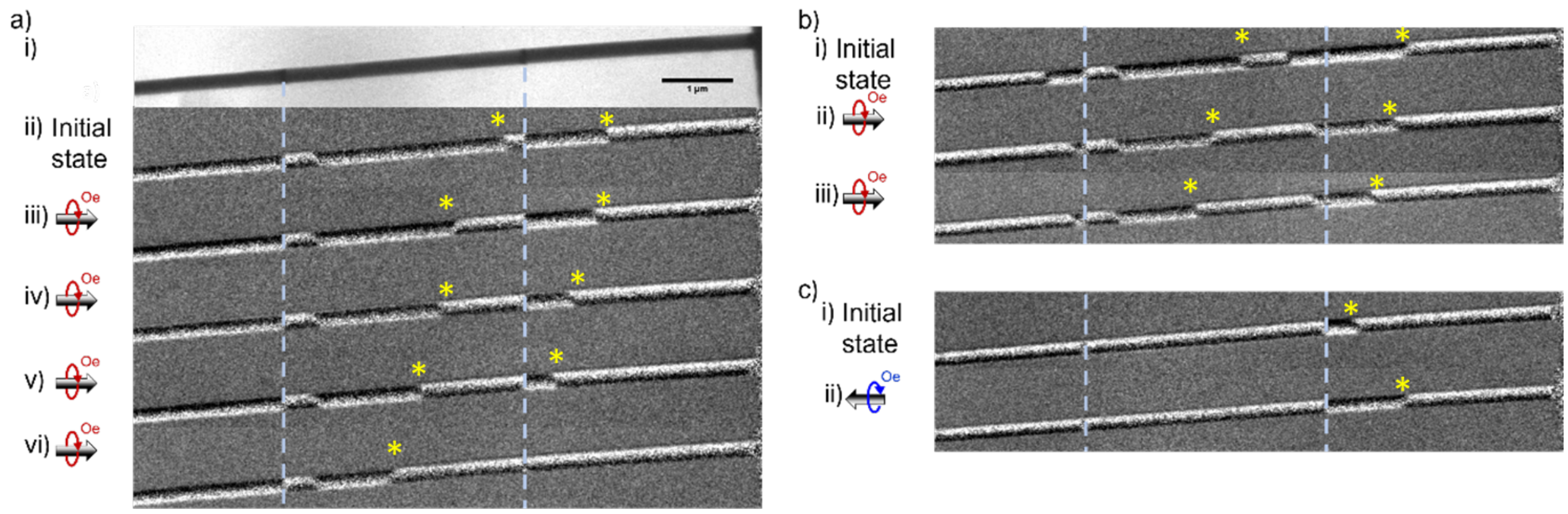

Figure S3: Transmission (a(i)) and XMCD (a(ii-vi), b(i-iii), c(i-ii)) images of a 220 nm nanowire. a) A 1.5 ns current pulse with positive polarity (i.e. with Oersted field favoring white-top and black-bottom domains) and a density of 5×10^{10} A/m^2 was injected between the acquisition of each image. b) A 1.5 ns current pulse with positive polarity and density 4×10^{10} A/m^2 injected between each image. c) A 1.5 ns current pulse with negative polarity (i.e. with Oersted field favoring black-top an white-bottom domains) and a density of 3×10^{10} A/m^2 was injected.

TEM Study: region of modulation 6

Figure S4 shows another example of crystallinity inhomogeneity along the nanowire, in this case around modulation 6. While the EDS maps indicate compositional homogeneity on both sides of the modulation, the ADF-STEM image suggests structural variations. It can be observed that on the left side of the modulation the nanowire exhibits a similar polycrystalline texture, whereas the right side shows larger grains or possibly a single-crystalline region. As can be seen in the XMCD image measured with TXM in the same region, the domain walls are pinned at these regions of crystallographic change.

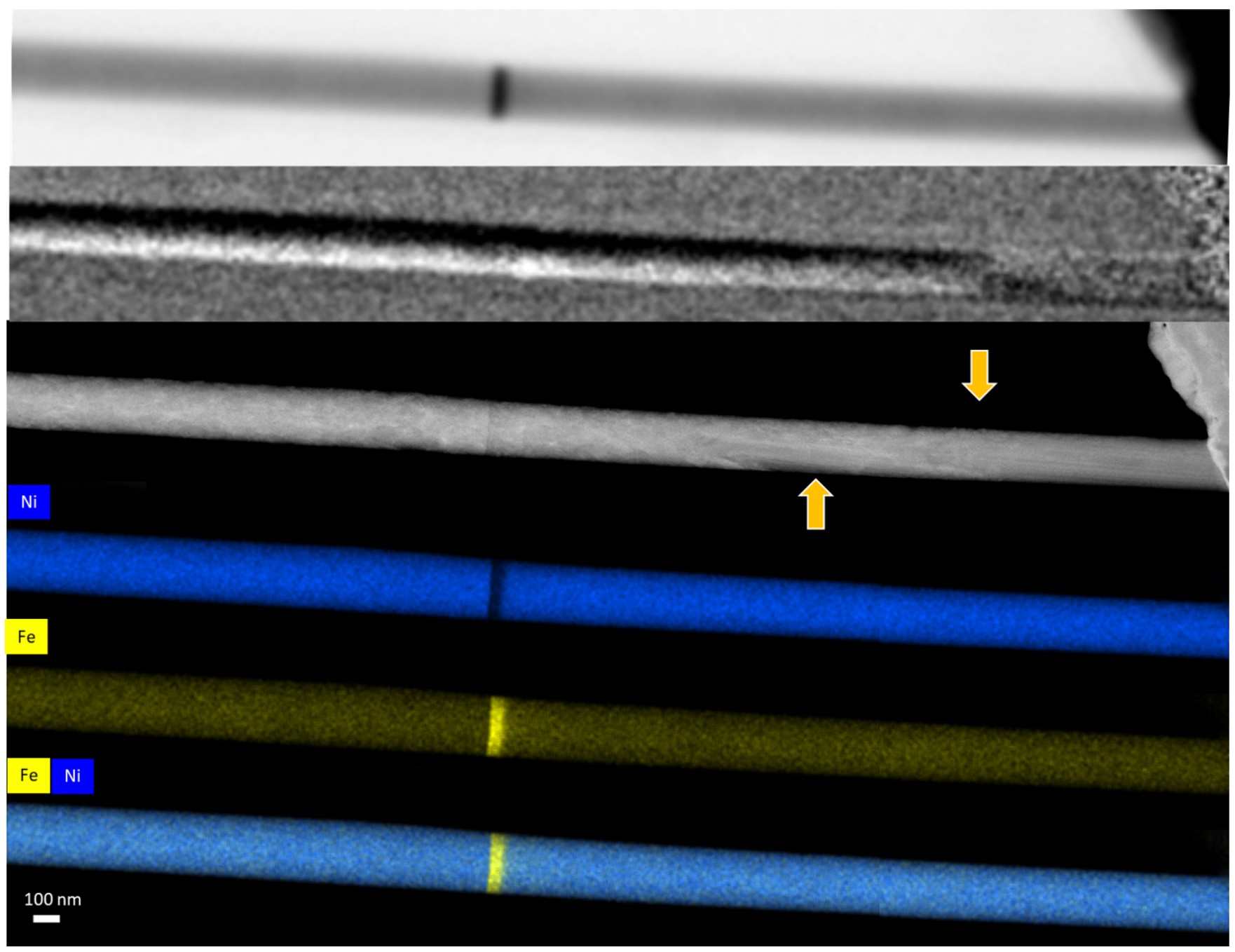

Figure S4: Stack of six images TXM, XMCD, continuous ADF-STEM images and corresponding EDS maps from the region of modulation 6. From top to bottom: ADF-STEM images, Fe and Ni net intensity maps, and a composite Ni + Fe intensity map. Arrows in the ADF-STEM image indicate large grains or single-crystalline regions.
